# Concordance of neonatal critical condition data between secondary databases: Florida and Texas birth certificate Linkage with medicaid analytic extract

**DOI:** 10.1186/s12874-023-01860-5

**Published:** 2023-02-20

**Authors:** Yasser Albogami, Yanmin Zhu, Xi Wang, Almut G Winterstein

**Affiliations:** 1 Department of Pharmaceutical Outcomes and Policy, College of Pharmacy, University of Florida, Gainesville, Florida USA; 2 Department of Clinical Pharmacy, College of Pharmacy, King Saud University, Riyadh, Saudi Arabia; 3 Center for Drug Evaluation and Safety, University of Florida College of Pharmacy, Gainesville, Florida USA

**Keywords:** Medicaid, Claims data, Birth certificates, Concordance, Neonatal complications, Measurement, Agreement, Sensitivity, Specificity, False positive

## Abstract

**Background:**

Limited information is available about neonates’ critical conditions data quality. The study aim was to measure the agreement regarding presence of neonatal critical conditions between Medicaid Analytic eXtract claims data and Birth Certificate (BC) records.

**Methods:**

Claims data files of neonates born between 1999–2010 and their mothers were linked to birth certificates in the states of Texas and Florida. In claims data, neonatal critical conditions were identified using medical encounter claims records within the first 30 days postpartum, while in birth certificates, the conditions were identified based on predetermined variables. We calculated the prevalence of cases within each data source that were identified by its comparator, in addition to calculating overall agreement and kappa statistics.

**Results:**

The sample included 558,224 and 981,120 neonates in Florida and Texas, respectively. Kappa values show poor agreement (< 20%) for all critical conditions except neonatal intensive care unit (NICU) admission, which showed moderate (> 50%) and substantial (> 60%) agreement in Florida and Texas, respectively. claims data resulted in higher prevalences and capture of a larger proportion of cases than the BC, except for assisted ventilation.

**Conclusions:**

Claims data and BC showed low agreement on neonatal critical conditions except for NICU admission. Each data source identified cases most of which the comparator failed to capture, with higher prevalences estimated within claims data except for assisted ventilation.

## Introduction

Randomized clinical trials tend to exclude vulnerable populations such as pregnant women and neonates, children under 30 days of age, for ethical reasons [1, 2], necessitating observational studies, using prospective cohorts or real-world data to be the cornerstone for perinatal drug safety and effectiveness assessments. Studies that assess maternal and/or neonatal health outcomes rely commonly on administrative data sources including Birth Certificates (BCs) and claims data [3–9].

BCs have been utilized widely for public health surveillance in maternal and neonatal epidemiology because they collect detailed information on delivery and the neonate’s condition in addition to information pertaining to pregnancy course and maternal characteristics. Claims data, on the other hand, provide rich longitudinal information on healthcare utilization that allows inferences about maternal clinical history and drug exposure before and during pregnancy and outcomes in mothers and infants after delivery. Because both data sources are not collected for research purposes, data quality is of concern. For studies that aim to make causal inferences about drug exposure and outcomes, misclassification bias, especially low specificity of outcome definitions [10], can bias the estimated exposure-outcome association. Accordingly, some studies have evaluated the quality of a number of variables in both BCs and claims data for multiple states [11–15], while others have used variables in BCs and/or claims data with unknown sensitivity (the ability to identify true cases) or specificity (the ability to identify true non-cases) [16–18].

Studies that have evaluated the validity of either BCs or claims data, usually against medical records, have centered around maternal variables including method of delivery, diabetes, hypertension, gestational age [19, 20], and major birth defects such as cleft palate or heart malformations [13, 15, 21]. Limited information is available about neonates’ critical conditions data quality such as birth injury or respiratory distress syndrome. This study aimed to evaluate the concordance between BCs and claims data on several neonatal critical conditions and quantified the extent of possible false positive cases in each data source.

## Methods

### Data Sources

Texas and Florida BC data from 1999–2010 were harnessed and deterministically linked to Medicaid claims data. BC data contain information on neonates’ demographics, date of birth, gestational age, weight, complications during labor, conditions and maternal data. The Medicaid Analytic eXtract (MAX) files include in- and outpatient encounter claims for neonates and their mothers in addition to pharmacy claims of dispensed prescriptions. We required that both neonates and their mothers be continuously enrolled in the insurance for at least 30 days postpartum to capture neonatal health information in claims data.

### BC and MAX linkage

**Fig. 1:**
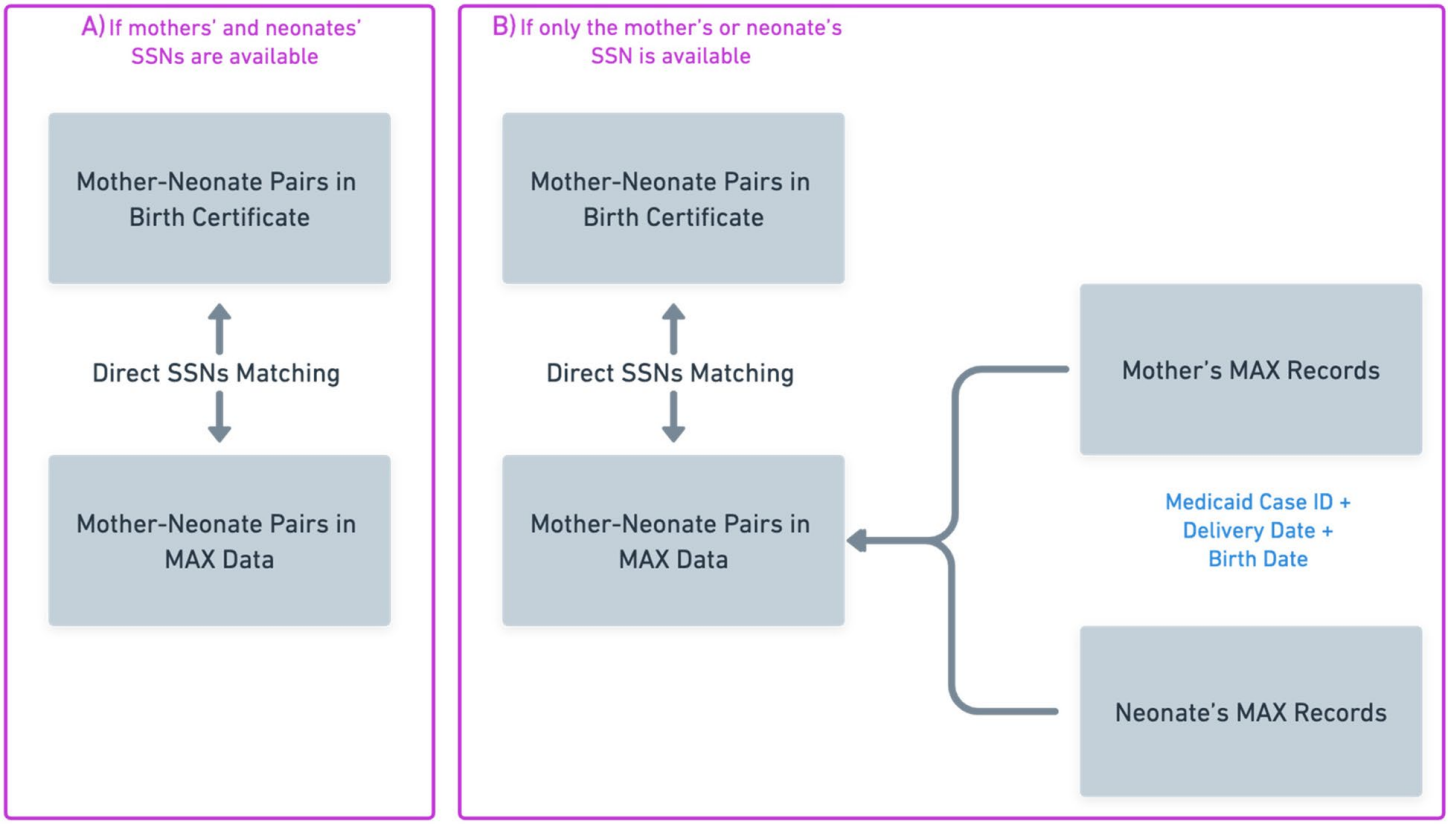
MAX and BC Linkage Flowchart

Mothers and neonates in MAX and BCs were linked using a two-step deterministic linkage procedure [22]. In brief, if both the mother’s and neonate’s Social Security Numbers (SSNs) were available for a mother-neonate pair in MAX and BC, both mothers and neonates were linked directly using exact SSN matching. If only the mother’s or neonate’s SSN was available, we employed an established linkage algorithm for both Texas and Florida [23]. We first linked neonates and the mothers in MAX using a Medicaid family identifier (i.e., the case ID) and delivery claim dates. Neonates’ birth date was then matched to delivery. The established algorithm was assessed for Texas and Florida showing > 99% of the positive predictive value (the proportion of true cases among total identified cases) [24]. After creating the mother-neonate pairs in MAX, the available SSN was then used to link the mother-neonate pairs in MAX to the BC (Fig. 1).

### Measurement of variables in MAX/BC

We measured the occurrence of birth injury, assisted ventilation (AV), seizure, respiratory distress syndrome (RDS) and neonatal intensive care unit (NICU) admission for each eligible neonate in both databases. In MAX data, the cases were ascertained based on the presence of at least one inpatient or outpatient encounter with condition-specific diagnosis codes in any diagnoses field within 30 days of birth on either neonates or mothers’ records using International Classification of Diseases, Ninth Revision, Clinical Modification (ICD-9-CM) or Current Procedural Terminology (CPT) codes (Table 1). The claims codes to identify NICU admission, RDS, AV and seizures have been previously validated against medical records and have shown high sensitivity and specificity or positive predictive value [14–16, 25, 26]. In BCs, clinical measurements are recorded manually in an official form which is subsequently entered in the birth certificate database. A certified registrars, midwives and healthcare providers obtain the clinical information, according to the institution’s policy [27, 28]. Therefore, a condition was flagged for a neonate if it was checked on the BC form, i.e., given value 1 in the database.

**Table 1:** ICD-9 & CPT Codes Used in MAX.

Variables	Type	Codes	Description	Validation
Neonatal Intensive Care Unit Admission (NICU Admission)	CPT	99,295	Initial inpatient neonatal critical care *(Deleted 01 Jan 2009)*	PPV = 92% (Medicaid Data [15])
	CPT	99,296	Subsequent inpatient neonatal critical care *(Deleted 01 Jan 2009)*	
	CPT	99,297	Subsequent inpatient neonatal critical care *(Deleted 01 Jan 2009)*	
	CPT	99,298	Subsequent intensive care *(Deleted 01 Jan 2009)*	
	CPT	99,299	Subsequent intensive care *(Deleted 01 Jan 2009)*	
	CPT	99,300	Subsequent intensive care *(Deleted 01 Jan 2009)*	
	CPT	99,468	Initial inpatient neonatal critical care, per day, for the evaluation and management of a critically ill neonate, 28 days of age or younger	
	CPT	99,469	Subsequent inpatient neonatal critical care, per day, for the evaluation and management of a critically ill neonate, 28 days of age or younger	
	CPT	99,477	Initial hospital care, per day, for the evaluation and management of the neonate, 28 days of age or younger, who requires intensive observation, frequent interventions, and other intensive care services	
	CPT	99,478	Subsequent intensive care, per day, for the evaluation and management of the recovering very low birth weight infant (present body weight less than 1500 g)	
	CPT	99,479	Subsequent intensive care, per day, for the evaluation and management of the recovering low birth weight infant (present body weight of 1500–2500 g)	
	CPT	99,480	Subsequent intensive care, per day, for the evaluation and management of the recovering infant (present body weight of 2501–5000 g)	
Respiratory Distress Syndrome (RDS)	ICD-9 dx	769.xx	Respiratory distress syndrome in newborn	PPV = 97% (Discharge Data [25]
Seizure	ICD-9 dx	779.0x	Convulsions in newborn	PPV = 86% (Medicaid data [37])
Assisted Ventilation (AV)	ICD-9 pc	96.7	Other Continuous Invasive Mechanical Ventilation	PPV = 93% (Discharge data [26])
	ICD-9 pc	96.70	Continuous Invasive Mechanical Ventilation of Unspecified Duration	
	ICD-9 pc	96.71	Continuous Invasive Mechanical Ventilation for Less Than 96 Consecutive Hours	
	ICD-9 pc	96.72	Continuous Invasive Mechanical Ventilation for 96 Consecutive Hours or More	
	ICD-9 pc	93.90	Non-Invasive Mechanical Ventilation	
Birth Injury	ICD-9 dx	767.xx	Birth trauma	NA

*ICD-9 PC* International Classification of Diseases, Ninth Revision, Clinical Modification (Procedure Codes)

*ICD-9 DX* International Classification of Diseases, Ninth Revision, Clinical Modification (Diagnosis Codes)

*CPT* Current Procedural Terminology

### Statistical analysis

Characteristics of the linked mother-neonate pairs in both states were examined in addition to the prevalence of each condition in each data source. We determined the level of agreement between data sources by calculating crude Kappa statistic. Unlike MAX where a missing diagnosis code is interpreted as absence of a condition, the BCs explicitly specify whether a condition was present or not, i.e., the BC forces selection of yes or no for a condition. Thus, we excluded BC records with missing determination of neonatal critical conditions, which occurred for less than 2% of records. Following usual convention, Kappa was categorized into high (> 80%), substantial (61%—80%), moderate (41%—60%), fair (21%—40%) and poor (≤ 20%) [29]. We also calculated the sensitivity of each data source as if the comparator were considered the gold standard in an attempt to quantify the ability of each source to capture cases identified by the other. For NICU admission, the analysis was conducted from 2004–2010 because this data field was added after 2003 as a result of a new BC form [30]. All analyses were performed using SAS 9.4 statistical software (SAS, Cary, NC). The study was approved by the University of Florida, Centers for Medicare and Medicaid Services, and Florida and Texas Departments of Health Institutional Review and Privacy Boards.

## Results

The study sample was 1,539,344 mother-neonate pairs after successful linkage of MAX and BCs. The sample included 558,224 and 981,120 mother-neonate pairs for Florida and Texas, respectively. The median age of the mothers was 23.7 in Florida and 22.9 in Texas (Table 2). Half of mothers who gave birth in Texas had at most graduated from secondary school. In contrast, mothers in Florida who had at least a high school diploma comprised almost three quarters. About 97% of deliveries occurred in medical facilities in Florida which was lower than in Texas (99.9%). The median number of prenatal visits was eleven in Florida which was slightly higher when compared to Texas. Preterm deliveries comprised 10.2% of linked pairs in Florida and 11.4% in Texas. Concordantly, we found higher prevalence of NICU admissions, RDS and AV in Texas compared to Florida regardless of data source (Table 3).

**Table 2:** Characteristics of mother and deliveries in the birth Certificate and Medicaid Analytic eXtract linked cohort

Characteristics	FLORIDA (*N* = 558,224)	TEXAS (*N* = 981,120)
Mother age (median, SD)^a^	23.7 (5.7)	22.9 (5.3)
Neonate's gender^a^		
Male	284,821 (51.0)	499,071 (50.9)
Female	272,798 (48.9)	482,049 (49.1)
Missing	605 (0.1)	0
Mother race (%)^a^		
White	231,583 (41.5)	261,530 (26.7)
Black	177,530 (31.8)	151,157 (15.4)
Hispanic	121,740 (21.8)	518,202 (52.8)
Others	6579 (1.2)	10,557 (1.1)
Missing	20,792 (3.7)	39,674 (4.0)
Mother education (%)^b^		
Secondary school or less	149,570 (26.8)	488,092 (49.7)
High school	245,514 (44.0)	255,125 (26.0)
Undergraduate	147,612 (26.4)	210,553 (21.5)
Graduate	4879 (0.9)	23,264 (2.4)
Missing	10,649 (1.9)	3636 (0.4)
Gestational Age (median, SD)^b^	39 (2.0)	39 (2.0)
Mother alcohol use (%)^b^		
Yes	2385 (0.4)	3533 (0.9)
No	549,170 (98.4)	372,618 (98.8)
Missing	6669 (1.2)	949 (0.3)
Mother tobacco use (%)^b^		
Smoking	76,689 (13.7)	45,397 (12.0)
Quitted	7158 (1.3)	NA^*^
Not smoking	472,943 (84.7)	330,816 (87.7)
Missing	1434 (0.3)	887 (0.3)
Birthplace (%)^b^		
Medical facility	540,183 (96.8)	980,267 (99.9)
Home	4450 (0.8)	727 (0.07)
Missing	13,591 (2.4)	124 (0.0)
Prenatal visit (median, SD)^b^	11 (3.8)	10 (4.0)
Preterm (%)^b^		
Yes	56,934 (10.2)	112,482 (11.4)
No	492,731 (88.3)	863,893 (88.1)
Missing	8559 (1.5)	4745 (0.5)
Birthweight (%)^b^		
Very low (< 1500 g)	6686 (1.2)	12,651 (1.3)
Low (1500 – 2499 g)	41,197 (7.4)	77,423 (7.9)
Normal (2500 – 3999 g)	472,673 (84.7)	841,045 (85.7)
High (> = 4000 g)	37,065 (6.6)	50,001 (5.1)
Missing	603 (0.1)	0

^a^data obtained from the Medicaid Analytic eXtract data

^b^data obtained from the Birth Certificate data

**Table 3:** Prevalence^a^ of neonatal critical conditions

**Neonatal critical condition**	FLORIDA		TEXAS	
	**MAX**	**BC**	**MAX**	**BC**
**Neonatal intensive care unit admission (NICU admission)**	7.40	6.31	10.00	7.05
**Respiratory distress syndrome (RDS)**	2.72	0.54	4.74	0.87
**Seizure**	0.24	0.02	0.25	0.02
**Assisted ventilation (AV)**	1.27	3.14	2.37	5.19
**Birth injury**	1.54	0.06	1.89	0.05

*MAX* Medicaid Analytic eXtract, *BC* Birth Certificate

^a^Prevalence was calculated as the number of identified cases per 100 neonate/mother pairs

**Fig. 2:**
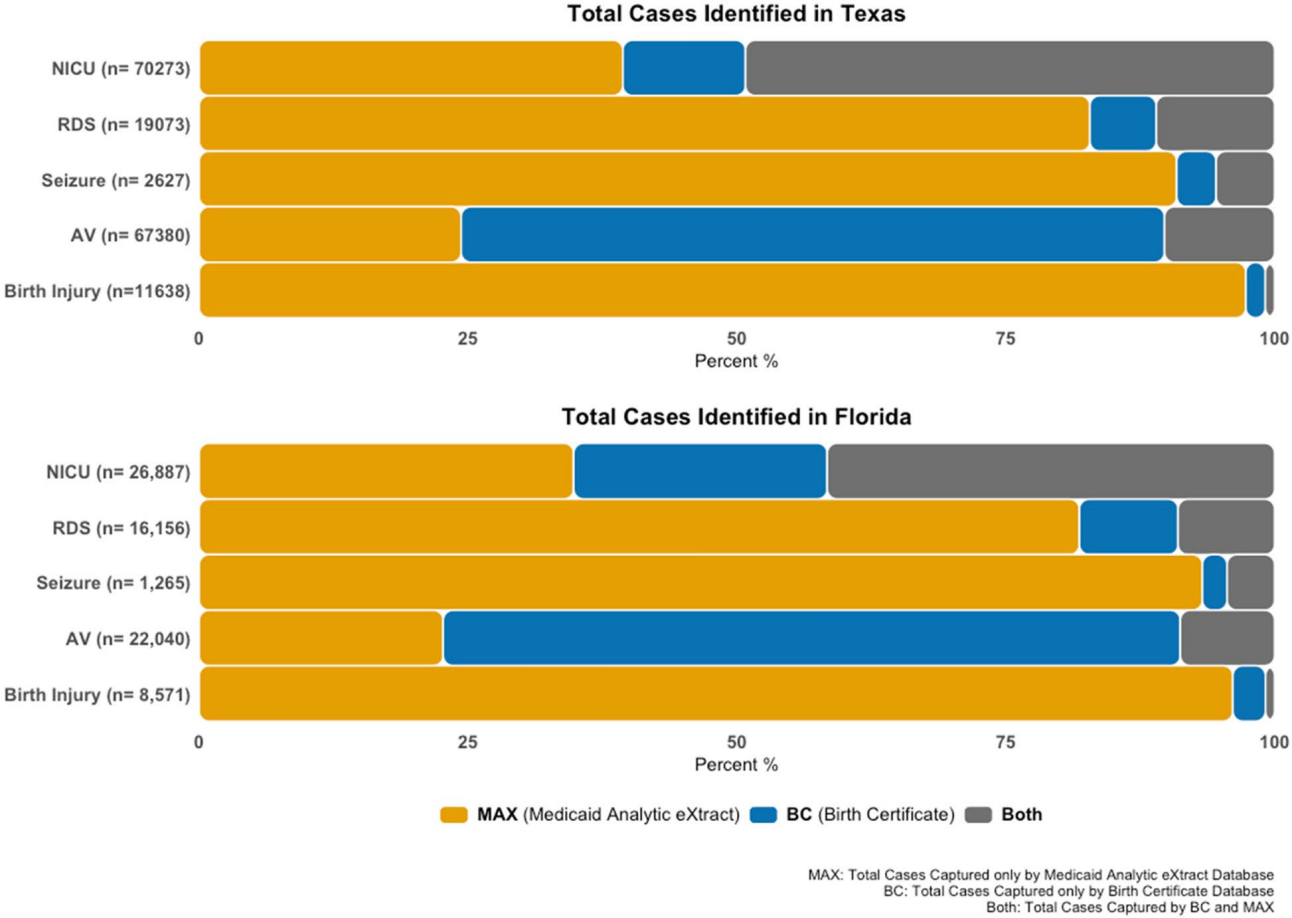
All cases distributed by data sources in Texas and Florida

The prevalence of neonatal critical conditions was consistently higher in MAX except for AV where the BC captured more than twice as many cases as MAX (Table 3). RDS showed a more than fourfold higher prevalence in MAX than in the BC and seizures were hardly ever captured in the BC. The highest absolute differences in prevalence between MAX and BC was for RDS (4%) in Texas. Figure 2 depicts the dissimilarity between MAX and BCs indicating that the majority of cases was predominantly identified by one data source but not the other. Except for NICU admission, more than 80% of all ascertained cases were captured by either MAX or BC but not both.

The agreement was moderate for NICU admission in Florida (Kappa = 56%) and reached the substantial level in Texas (63%). For respiratory distress syndrome and assisted ventilation, Kappa showed poor agreement between BC and MAX in Texas (19% and 16%) and Florida (16%-15%). There was 8%—10% agreement between MAX and BC for seizures in the two states (Table 4). Birth injury showed extremely low agreement (Kappa 1.5%). Kappa values were generally slightly lower for Florida than Texas with MAX identifying lesser cases captured by the BC, but the BCs of both states showing similar sensitivity to capture cases in MAX.

**Table 4:** Estimated Kappa and sensitivity

State	Neonatal Critical Condition	MAX	BC	Both	Kappa	MAX Sensitivity^a^	BC Sensitivity^b^
**FL**	**Neonatal intensive care unit admission (NICU admission)**	20,548	17,524	11,185	55.7	0.64	0.54
	**Respiratory distress syndrome (RDS)**	14,673	2,933	1,450	15.7	0.49	0.10
	**Seizure**	1,236	85	56	8.4	0.66	0.05
	**Assisted ventilation (AV)**	6,933	17,039	1,932	14.5	0.11	0.28
	**Birth injury**	8,311	333	73	1.5	0.22	0.01
**TX**	**Neonatal intensive care unit admission (NICU admission)**	62,258	42,592	34,577	62.8	0.81	0.56
	**Respiratory distress syndrome (RDS)**	17,897	3,276	2,100	18.6	0.64	0.12
	**Seizure**	2,531	239	143	10.2	0.60	0.06
	**Assisted ventilation (AV)**	23,311	50,969	6,900	15.8	0.14	0.30
	**Birth injury**	11,427	312	101	1.6	0.32	0.01

*BC* Birth Certificate, *FL* Florida, *TX* Texas, MAX: Medicaid Analytic eXtract;

^a^Ability of MAX to capture all cases identified by BC (Cases identified by both / all cases in BC)

^b^Ability of BC to capture all cases identified by MAX (Cases identified by both / all cases in MAX)

Assuming that neither data source captured false positives, we found generally better sensitivity for MAX to capture BC cases than vice versa: MAX captured > 80% of NICU admissions that were recorded in Texas and 64% in Florida, while the BC only captured about half of all NICU admissions in MAX (Table 4). The only exception was AV, with about 70% of all identified assisted ventilation cases recorded on the BC and better capture of MAX cases in the BC (about 30%) than vice versa (about 10%).

## Discussion

Our comparison of birth certificate and Medicaid claims data in measuring neonatal critical conditions in two large states found large variation in concordance between the two data sources. Capture of NICU admission showed moderate to substantial agreement between BCs and MAX, while variables such as birth injury and seizures had extremely low agreement. Importantly, except for NICU admission, 70% or more percent of cases in MAX were not captured by BCs in either state. MAX found higher prevalences for all examined conditions except for need for assisted ventilation with more than twice as many cases identified in the BC.

The overall higher prevalences captured by MAX may be explained by either lower sensitivity of data capture in BCs or false positive cases captured in MAX. Considering our use of previously validated claims-based algorithms, suggesting PPV >  = 86% for all evaluated conditions, low specificity of MAX-derived cases is unlikely to explain the lower prevalences reported in BCs. This conclusion is supported by a recent evaluation of BC data by the Centers for Disease Control and Prevention (CDC), which found quality to vary across states and hospitals [31]. The study, limited to two unidentified states and only 8 hospitals, reported agreement for NICU admissions and need for AV to be substantial or high for one state and low to extremely low for the other. The study confirmed that especially the sensitivity of BC is low, which reiterates previous findings that highlight underreporting of medical conditions as a key limitation of BCs [28, 32–34]. Specific to seizures, one research group studied neonatal seizure by evaluating capture on the BC [14] against hospital discharge and Medicaid claims [35]. Consistent with our finding, the authors reported low Kappa estimates (9–12%), which they attributed to BC underreporting. Of note, the timing of BC completion relative to the occurrence or discovery of neonatal conditions may further reduce BC sensitivity.

Failure of MAX data to capture cases detected by the BC on the other hand, may be explained by erroneous data on the BC or limited sensitivity of MAX. The previously quoted CDC study found in one state that the proportion of false positives on the BC varied between 15–30% for AV (depending on definition) and 11% for NICU admission. A more recent report from five hospitals in New York city shows a similar pattern of false-positive AV cases [36]. suggesting that BCs may not only lack sensitivity but also have some specificity issues in correctly identifying neonatal conditions [26]. Similarly, Zollinger et al [11] compared Indiana BC data to medical records for a variety of variables including RDS, AV, seizure and birth injury. The corresponding results showed extremely low specificity of BCs in identifying RDS, AV, seizure and birth injury cases. Administrative claims databases, on the contrary, have been compared to medical records for some variables and concluded overall high specificity. Two studies assessed ICD-9-CM codes for neonatal mechanical ventilation against medical records resulting in specificity of 99.7% and 97.1% in Canadian and Australian discharge databases, respectively [25, 26]. The same Australian discharge database showed 92.4% specificity for RDS. In addition, Bateman et al [37] validated neonatal seizure codes in MAX data and found a high positive predicative value of 86%. Although some of these validation studies occurred outside the U.S. and may not be generalizable to local coding conventions, the similarity in findings regarding specificity issues in BCs raises concerns about bias when used in inferential analyses. It should be noted that claims data’s focus on billing of medical services may result in varying sensitivity by prioritizing those diagnoses that are relevant for reimbursement decisions.

Our findings, taken together, support the assumption that MAX data may produce superior sensitivity and specificity in capturing the evaluated neonatal conditions. An exception of this observation appears to be in the measurement of need for assisted ventilation, which may be inherent in coding practices for billing purposes. For example, short-term need for assisted ventilation immediately after delivery, which is captured on the BC, may be incorporated in capitated billing arrangements and thus not appear itemized in billing data.

Motivation or incentives as well as training may influence the quality of data and discrepancy between BC and MAX. Both CDC and the American College of Obstetricians and Gynecologists have also pointed to lack of standardization of obstetric clinical data definitions [31, 38]. Ongoing efforts to improve BC documentation via enhanced training and standardization warrant future studies as more recent MAX and BC data are made available.

Although our results only cover two states and are therefore not nationally representative, Texas and Florida supply a large portion of infant information to the national surveillance systems. Thus, national trends of neonatal critical conditions such as seizures that are estimated from BCs ought to be interpreted with caution. Moreover, causal inference research that use BC neonatal critical conditions should interpret the results with extreme caution given the high false positive rates of the BC data. Claims data sources appear to be more suited for causal inference research studying the neonatal critical conditions.

In conclusion, we compared the extent of agreement between BCs and claims data regarding capture of neonatal critical conditions in two large states and found low agreement. Both data sources captured cases that the other one did not, presumably due to underreporting or capture of false positives. Future research ought to examine reasons for discrepancies between the two data sources.

## Data Availability

The data that support the findings of this study are available from the Texas State Department of Health and the Florida State Department of Health but restrictions apply to the availability of these data, which were used under license for the current study, and so are not publicly available. Data are however available from the authors upon reasonable request and with permission of the State Departments of Health in Texas and Florida.
